# Potential Antioxidative Components in *Azadirachta indica* Revealed by Bio-Affinity Ultrafiltration with SOD and XOD

**DOI:** 10.3390/antiox11040658

**Published:** 2022-03-29

**Authors:** Min-Xia Fan, Gui-Lin Chen, Ming-Quan Guo

**Affiliations:** 1 Key Laboratory of Plant Germplasm Enhancement and Specialty Agriculture, Wuhan Botanical Garden, Chinese Academy of Sciences, Wuhan 430074, China; fanminxia@wbgcas.cn (M.-X.F.); glchen@wbgcas.cn (G.-L.C.); 2 Sino-Africa Joint Research Center, Chinese Academy of Sciences, Wuhan 430074, China; 3 Innovation Academy for Drug Discovery and Development, Chinese Academy of Sciences, Shanghai 201203, China

**Keywords:** *Azadirachta indica*, ultrafiltration liquid chromatography, mass spectrometry, antioxidation, superoxide dismutase, xanthine oxidase, molecular docking

## Abstract

*Azadirachta indica* (*A. indica*) has been widely used due to its diverse pharmacological activities. However, there are currently few studies on its responsible antioxidant ingredients against superoxide dismutase (SOD) and xanthine oxidase (XOD). In this study, the antioxidant activities of *A. indica* were evaluated by a 2,2′-azinobis-(3-ethyl-benzthiazoline)-6-sulfonic acid) and ferric-ion-reducing antioxidant power method. Meanwhile, total polyphenol and flavonoid content were determined to reveal that they were the highest in ethyl acetate (EA) fraction. Next, compounds with the most antioxidant activity were screened out from EA fraction by bio-affinity ultrafiltration liquid chromatography–mass spectrometry (UF-LC-MS) with SOD and XOD. As a result, gallic acid, protocatechuic acid and (−)-epicatechin were identified as potential SOD ligands with relative binding affinity (RBA) values of 2.15, 1.78 and 1.61, respectively. Additionally, these three ligands could effectively interact with SOD in molecular docking with binding energies (BEs) ranging from −3.84 ± 0.37 to −5.04 ± 0.01 kcal/mol. In addition, carnosic acid exhibited a strong binding affinity to XOD with an RBA value of 2.05 and BE value of −8.24 ± 0.71 kcal/mol. In conclusion, these results indicated that *A. indica* might have good antioxidant activity and antigout potential, and the UF-LC-MS method is suitable and efficient for screening both SOD and XOD ligands from *A. indica*.

## 1. Introduction

*Azadirachta indica* (*A. indica,* neem), belonging to the genus *azadirachta* (Meliaceae), is a typical, tropical–subtropical, multipurpose, traditional, medicinal plant [1]. It was reported as a tree to solve global problems by the US National Academy of Science, and the United Nations declared it as “Tree of the 21st century” [2,3]. *A. indica* is a fast-growing evergreen plant with white honey-scented flowers and oval fruit with brown seeds. The different parts of this magical tree, including bark, roots, seeds, fruits, leaves, and flowers, are extensively used for different purposes in the treatment of ulcers, gastrointestinal problems, diabetes, infectious diseases, etc. [2,4,5]. In addition, *A. indica* has extensive pharmacological activities such as antihyperglycemia, antimalaria, antiulcer, diuretic, antibacterial, antiviral, antioxidant, liver protection, anticancer, antiparasitic, insecticidal activities. Moreover, it can be used for antipyretic, nervous, antianxiety, analgesia and cardiovascular protection [6,7,8]. *A. indica* gained lots of attention, and more than 300 compounds were isolated and identified in this plant, which are the chemical basis for its multidirectional activities [2]. These compounds were classified into isoprenoids and non-isoprenoids [9,10,11], and the isoprenoids include diterpenes, triterpenes, gedunin and its derivatives, vilasinin-type compounds and C-secome-liacins, while the non-isoprenoids include proteins (amino acids), carbohydrates (polysaccharides), sulfur-containing compounds, polyphenols, flavonoids and their glycosides, coumarins and tannins, and aliphatic compounds [2,9,12]. Thus, it can be seen that the compounds of *A. indica* are structurally complex and chemically diverse, and they are known as the storehouse of phytochemicals. However, when it comes to revealing the responsible compounds for a certain pharmaceutical activity, such as antioxidant compounds in this plant, the traditional phytochemical method is time-consuming and laborious. To overcome the disadvantages of traditional methods, bio-affinity ultrafiltration coupled to liquid chromatography–mass spectrometry (UF-LC-MS) has been actively developed, combining drug-targets-based affinity ultrafiltration and LC-MS to simultaneously screen out and identify small-molecule ligands from a complex mixture of medicinal plants, and UF-LC-MS has demonstrated the advantages of rapid screening, high throughput, high specificity and fewer samples [13,14], which are widely used methods in the screening of active ingredients.

Reactive oxygen species (ROS) are necessary for a number of important life activities, including cellular growth, proliferation and differentiation, energy supply, health problems and the aging of some organisms. Meanwhile, ROS are also unavoidable toxic byproducts of aerobic metabolism, causing damage to cellular components, such as proteins, DNAs and lipids that could lead to cell dysfunction and even apoptosis. Therefore, ROS are supposed to have a dual effect in biological processes [15,16], and they are constantly produced and eliminated by the antioxidant system in the body. Once this equilibrium is disturbed, some ROS cannot be removed in time, and the accumulated ROS will cause certain damage to the organism, such as oxidative stress damage. In order to prevent the damage of oxygen free radicals to the cell body, almost all cells have a subtle protective system to remove all kinds of ROS produced by cell metabolism. During these processes, superoxide dismutase (SOD) plays an important fundamental role in protecting cells from ROS as a powerful natural antioxidant enzyme. It can convert the superoxide (O_2_^•−^) to hydrogen peroxide (H_2_O_2_), then be converted into harmless water (H_2_O) with the help of other enzymes in the oxidation process [17]. Xanthine oxidase (XOD) catalyzes the oxidative hydroxylation reaction of hypoxanthine and xanthine, and reduces oxygen in the center of xanthine to generate reactive oxygen. Excessive XOD activity will lead to an increase in H_2_O_2_ and O_2_^•−^, increasing the possibility of oxidative stress damage generated by ROS in the enzymatic process. In addition, XOD is also a drug target for the treatment of gout, which is often accompanied by oxidative damage in patients with gout. Therefore, XOD is an effective target for the treatment of diseases related to oxidative tissue damage, and the inhibition of this enzymatic pathway would be beneficial to some certain diseases such as gout [18]. Thus, it is promising to screen antioxidant compounds from *A. indica* to solve oxidative damage and maintain the balance between oxidation and antioxidant based on two closely related enzymes, i.e., SOD and XOD. At present, studies on screening antioxidant active ingredients from *A. indica* by UF-LC-MS with SOD and XOD as target enzymes have not been reported.

In this context, the active fractions should firstly be screened and selected due to the chemical complexity and diversity of *A. indica*. Thus, ABTS (2,2′-azinobis-(3-ethyl-benzthiazoline)-6-sulfonic acid) and FRAP (ferric-ion-reducing antioxidant power) assays were used to evaluate the antioxidant capacities of *A. indica*, including ethanol crude extract (CE), n-hexane (n-Hex), ethyl acetate (EA), n-butyl alcohol (n-BuOH) and aqueous (WA) fractions. Then, the total phenolic and flavonoid content (TPC, TFC) were carried out to reveal their correlation to the antioxidant activities. Furthermore, UF-LC-MS with SOD and XOD as targets enzymes was used to rapidly screen antioxidant active ingredients from the EA fraction of *A. indica*. Finally, molecular docking was applied to decipher the interactions between the active ingredients and the target enzymes. This work aims to explore the most potent antioxidant components in *A. indica*, which could provide valuable information in order to promote the development and utilization of *A. indica* as a potential natural antioxidant and antigout agent.

## 2. Materials and Methods

### 2.1. Plant Materials

*A. indica* root bark was collected from Mount Kenya and authenticated by Professor Guangwan Hu, a senior taxonomist from the Key Laboratory of Plant Germplasm Enhancement and Specialty Agriculture (Wuhan Botanical Garden), Chinese Academy of Sciences. The specimens were preserved in the herbarium of the Key Laboratory of Plant Germplasm Enhancement and Specialty Agriculture with the voucher specimen numbers (No. 2020-0021). Dried root barks of *A. indica* were powdered and extracted with 95% ethanol (3 times, 2 days each time) at room temperature to obtain CE (698.9 g) and its subsequent partition fractions, including n-Hex (80.3 g), EA (157.0 g), n-BuOH (290 g) and WA (123 g) fractions. The obtained samples were dried and stored at 4 °C for later use.

### 2.2. Chemicals and Reagents

Rutin, gallic acid, Folin–Ciocalteu reagent, 1,3,5-tri(2-pyridyl)-2,4,6-triazine (TPTZ), 2,2′-azinobis-(3-ethylbenzthiazoline-6-sulfonic acid) (ABTS), ascorbic acid (vitamin C, Vc), 6-hydroxy-2,5,7,8-tetramethylchroman-2-carboxylic acid (Trolox) and butylated hydroxytoluene (BHT) were purchased from Sigma-Aldrich Corp. (St. Louis, MO, USA). The purity of the standard substance involved in the experiment was above 95%. The acetonitrile (ACN) and methanol of HPLC grade were supplied by TEDIA Company Inc. (Fairfield, OH, USA). Other chemicals and solvents of analytical grade were acquired from Shanghai Chemical Reagent Corp. (Shanghai, China). Superoxide dismutase (SOD) and xanthine oxidase (XOD) were bought from Shanghai Yuanye Bio-Technology Co., Ltd. (Shanghai, China). Ultrafiltration device with pore sizes of 30 kDa (YM-30) was purchased from Millipore Co., Ltd. (Bedford, MA, USA). All aqueous solutions of ultra-pure grade for HPLC and HPLC-UV-ESI-MS/MS analyses were prepared with a Milli-Q System (18.25 MΩ, Millipore, Billerica, MA, USA).

### 2.3. Instruments

HPLC-UV/ESI-MS/MS was conducted with a Thermo Accela 600 series HPLC system coupled with a TSQ Quantum Access MAX mass spectrometer (Thermo Fisher Scientific, San Jose, CA, USA). Ultra-performance liquid chromatography–quadrupole-time of flight mass spectrometry (UPLC-Q-TOF-MS/MS) was carried out with an Agilent 1290 L UPLC coupled to Agilent 6530 MS (Agilent Technologies, Santa Clara, CA, USA) with Sunniest C18 HT (2.1 mm × 100 mm, 2 µm). As for the HPLC analysis, an Agilent 1220 LC (Santa Clara, CA, USA) with a RP-C18 column (Waters Symmetry RP-C18, 4.6 mm × 250 mm, 5 µm) was applied, and the UV absorbance was recorded by UV/VIS Spectrophotometer (UV1100, Shanghai, China). Centrifugation of samples was carried out by low-temperature high-speed centrifuge. 

### 2.4. Determinations of Antioxidant Activity of A. indica

#### 2.4.1. ABTS•^+^ Radical Cation Scavenging Activity Assay

ABTS•^+^ radical cation scavenging activity was assessed using the reported method with slight modifications [19]. In brief, the ABTS solution (7 mM in H2O) was mixed with phosphate-buffered saline (pH 7.4) and then diluted with MeOH to obtain ABTS•^+^ stock solution with an absorbance of 0.700 ± 0.100 at λ = 734 nm. Then, 10 µL of samples, appropriately diluted with methanol, was added to 190 µL of ABTS•^+^ solution and gently shaken. The mixture was incubated for 6 min in darkness. Vitamin C (Vc) and Trolox were used as positive controls. All samples and controls were tested in triplicate (*n* = 3). The ABTS•^+^ free-radical scavenging activity was determined as the formula: scavenging rate (%) = [(Acontrol − Asample)/Acontrol] × 100%, where Acontrol and Asample represent the absorbance of the control and sample group, respectively. The final results were expressed as scavenging rate (%) and IC50 values. The IC50 value represents the 50% inhibition ratio of ABTS•^+^ activity.

#### 2.4.2. Ferric-Ion-Reducing Antioxidant Power Assay (FRAP)

The FRAP assay on ethanol crude extract and its four partition fractions was performed using the method by Benzie and Szeto [20]. The FRAP reagent was a mixture of 300 mM acetate buffer (pH 3.6), 10 mM TPTZ solution and 20 mM FeCl_3_·6H_2_O in a ratio of 10:1:1 (*v/v/v*) and was heated for 10 min (37 °C). Appropriately diluted samples were mixed with fresh FRAP and incubated at 37 °C for 10 min. The absorbance was recorded at 593 nm by triplicate tests (*n* = 3). FeSO_4_·7H_2_O was used to establish calibration curve with positive controls (Vc, BHT). The FRAP activity assay was expressed as mg Fe^2+^/g of the sample tested.

### 2.5. Determination of Phenolic Constituents

#### 2.5.1. Total Phenolic Content (TPC)

The TPCs of CE, n-Hex, EA, n-BuOH and WA were achieved by the Folin–Ciocalteu method [21] with slight modifications. In short, 200 µL properly diluted sample solution was mixed with Folin–Ciocalteu phenol reagent (1000 µL) and incubated for 3 min. After that, 800 µL of sodium carbonate (15%, *w/v*) solution was added to the mixture and incubated for 1 h in the dark. Gallic acid (GA) was used as a standard, and the UV was recorded at λ 760 nm. The TPC was expressed as milligram GA equivalents (GAE) per gram of the sample (mg GAE/g).

#### 2.5.2. Determination of Total Flavonoid Contents (TFC)

The TFCs of CE, n-Hex, EA, n-BuOH and WA fractions were estimated (*n* = 3) by the colorimetric assay [19]. Adequately diluted (2 mL) sample was mixed with distilled water (3 mL) and 500 µL of NaNO_2_ (5%, *w/v*) solution. After incubation for 6 min, AlCl_3_ solution (10%, *w/v*, 500 µL) was added and incubated for a further 6 min. Then, 4% NaOH solution (4 mL) was added to the mixture solution and incubated for 15 min. The absorbance was read at 510 nm by UV/VIS spectrophotometer (UV-1100, MAPADA, Shanghai, China). Rutin was used as the standard, and the results are expressed as milligrams of rutin equivalent (RE) per gram of dry weight of the sample (mg RE/g dry weight). 

### 2.6. Sample Preparation and Screening of the Potential Ligands of SOD and XOD with UF-LC-MS

Firstly, EA fraction (10 mg) of *A. indica* was dissolved with Tris-HCl (pH = 7.8, 995 µL with 5 µL DMSO) buffer solution and ultrasonicated for 30 min, which was used as tested sample solution. The UF-LC-MS procedures for screening potential antioxidant active components from EA fraction with high relative binding affinity to SOD and XOD were carried out depended on previous studies [13,22,23,24]. Briefly, 80 µL of sample solution (10.0 mg/mL) was incubated with SOD (0.2 U/µL) or XOD (0.25 U/µL) at 37 °C in the dark for 1 h. The incubated solutions were transferred into 30 KDa cut-off ultrafiltration membranes and centrifuged at 10,000 rpm for 10 min at 25 °C to remove the unbound ligands, and the nonspecific ligands were further removed by washing with Tris-HCl solution (pH 7.8, 200 µL) three times through centrifugation. Then, the ligands with specific binding to SOD or XOD were released by incubating with methanol (10% aqueous) for 10 min and centrifuging for 10 min (*n* = 3). Finally, the ligands solution was dried and reconstituted with 50 µL 90% aqueous methanol for further analysis. In addition, the inactive enzyme group was set up, and the treatment method is consistent with the active enzyme group. 

### 2.7. UPLC-Q-TOF-MS/MS Analysis

UPLC-Q-TOF-MS/MS analysis of EA fraction from *A. indica* was performed by ultra-high-performance liquid chromatography (UPLC) coupled to quadrupole-flight mass spectrometry (Q-TOF-MS/MS, Agilent 1290 L, Agilent 6530 MS, Agilent Technologies, Santa Clara, CA, USA). A Sunniest C18 HT (2.1 mm × 100 mm, 2 µm) column was used for the chromatographic separation at 25 °C. The Q-TOF-MS analysis was conducted with a dual ESI source in the negative-ion modes. The ultrapure water (A, 0.1% formic acid) and acetonitrile (B, ACN) were used, and the flow rate was 0.2 mL/min. The injection volume was 20 µL and the LC elution gradient was set as follows: 0–3 min, 5–10% B; 3–8 min, 10–10% B; 8–20 min, 10–23% B; 20–30 min, 23–30% B; 30–40 min, 30–60% B; 40–45 min, 60–95% B. The MS parameters were set as follows: the capillary voltage (Vcap) was 3500 V and fragmentor voltage was 175 V. The capillary temperature and drying gas flow rate were 350 °C and 8 L/min, respectively. The nebulizer pressure was set at 35 psi. The fixed collision energies were set as 10, 20, and 40 V. Mass Hunter workstation (Agilent) with a mass range of *m/z* 100–1100 at a rate of 1 spectra per second was utilized to obtain profile data. Compounds were identified by comparing their retention time, parent ions and mass fragments with references and databases.

### 2.8. Molecular Docking Study

The interaction mechanism between ligands and enzymes was further explored by molecular docking with AutoDock tools-1.5.6 software and AutoDock4 software (Scripps Research Institute, San Diego, FL, USA) based on previous studies [25,26]. The simulation was conducted with crystal structure of copper/zinc SOD (PDB 1CBJ) [27] and XOD (PDB 1FIQ), in which 2-hydroxybenzoic acid was selected as the active site and then removed [28,29]. Their crystallized structures were downloaded from RSCB Protein Data Bank (http://www.rcsb.org/pdb, 22 February 2022), and the 3D structures of the ligands were built by ChemBio3D Ultra 14.0 (CambridgeSoft Corp., Cambridge, MA, USA). The SOD and XOD structure were optimized by Discovery Studio 4.5, and the grid box of the SOD was 60 × 60 × 60 grid points with a center grid box of X = 3.919000, Y = 19.127625 and Z = 43.770675. Meanwhile, the grid box of the XOD was 60 × 60 × 60 grid points, and the center grid box was X = 26.543600, Y = 10.161800 and Z = 113.364500. Autogrid4 and AutoDock4 were run, and the root-mean-square deviation (RMSD) was evaluated to validate the docking parameters and the stability of enzymes and ligands in the simulated system [30].

### 2.9. Validation of Potential Ligands Activity by UF-LC-MS

In order to further verify the affinity between the potential ligands mentioned above and the enzyme, we used UF-LC-MS to determine the relative IC_50_ of the potential ligands. The method of UF-LC-MS was the same as above. For SOD, quercetin (IC_50_ = 0.58 mM to SOD), which is known to have significant antioxidant activity, was selected as the positive control [31]. Quercetin and potential ligands of SOD were performed with UF-LC-MS to obtain their respective RBA values, and then the relative IC_50_ of potential ligands was obtained based on relative RBA (Figure S1 and Table S1).

### 2.10. Statistical Analysis

All data in this work are expressed as mean ± standard deviation (SD) of triplicate experiments. The percentages of scavenging rates or the inhibition rates are plotted. IC_50_ values were obtained by plotting the percentages of scavenging rates or the inhibition rates against the sample concentrations (six different concentration gradients in triplicate). *p* < 0.05 was considered to be statistically significant.

## 3. Results and Discussion

### 3.1. Evaluation of Antioxidant Activities

**Figure 1:**
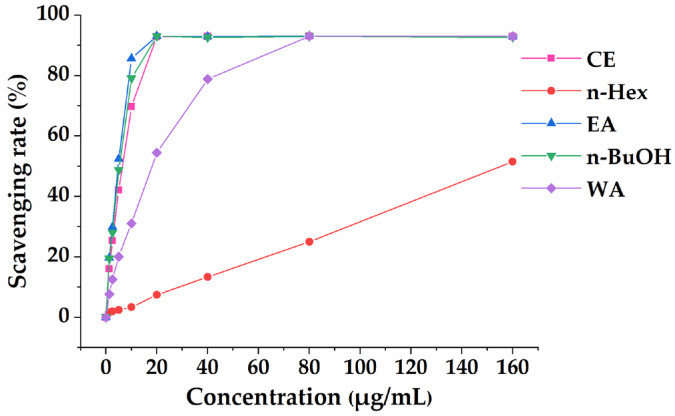
The scavenging rate (%) of ABTS by CE, n-Hex, EA, n-BuOH and WA fractions of *A. indica*. All of the values are expressed as means (%) ± SD of triplicated experiments.

**Figure 2:**
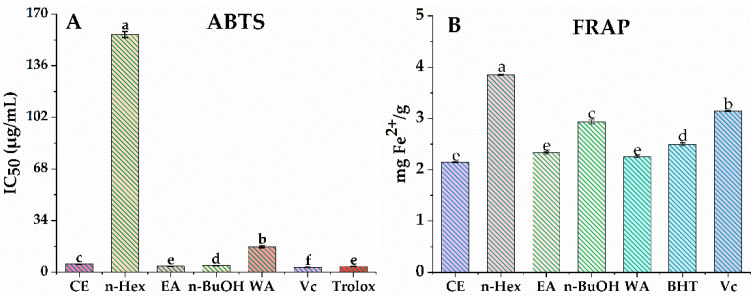
Antioxidant activity of CE, n-Hex, EA, n-BuOH and WA fractions of *A. indica.* (**A**) the IC_50_ value of ABTS radical scavenging assay, (**B**) ferric-ion-reducing antioxidant power (FRAP) assay. Mean values with different letters (a–f) were significantly different at a level of *p* < 0.05 (*n* = 3) by DMRT (Duncan’s multiple range test).

The antioxidant potentials of CE, n-Hex, EA, n-BuOH and WA of *A. indica* were evaluated and compared using two representative assays (ABTS and FRAP) in parallel because of the different scavenging modes of ROS and the complexity of natural phytochemicals [19]. Figure 1 demonstrates that the CE and its four fractions had a certain scavenging effect on ABTS free radicals in a dose-dependent manner. As shown in Figure 2A, EA fraction showed notable activities in ABTS radical scavenging assay with an IC_50_ value of 3.95 ± 0.08 µg/mL, compared to the positive controls of Vc and Trolox with IC_50_ values of 3.22 ± 0.04 µg/mL and 3.87 ± 0.15 µg/mL, respectively. The n-Hex fraction displayed the lowest ABTS free radical scavenging ability with IC_50_ = 156.48 ± 2.15 µg/mL among other fractions. The results of the FRAP assay (Figure 2B) indicated that the CE (2.15 mg Fe^2+^/g) exerted a similar iron reduction ability in comparison to EA fraction (2.33 mg Fe^2+^/g) and WA fraction (2.26 mg Fe^2+^/g). The n-Hex fraction (3.86 mg Fe^2+^/g) displayed a relatively higher iron reduction ability, compared with the positive controls of BHT with 2.50 mg Fe^2+^/g and Vc with 3.14 mg Fe^2+^/g. Figure 2A,B show that *A. indica* exhibited a notable antioxidant activity through ABTS and FRAP assays; especially EA fraction had the highest scavenging effect on ABTS free radicals and showed a relatively prominent iron reduction ability. Hence, we chose the EA fraction for further research.

### 3.2. Total Phenolic and Flavonoid Content

Numerous studies have shown that polyphenols and flavonoids in plants are natural antioxidants, and they can exert antioxidant effects through the strong capture of free radicals such as ROS. It was found that *A. indica* possessed a good antioxidant activity, and its EA fraction had the highest scavenging effect on ABTS free radicals. In order to further explore the potential antioxidant compounds in *A. indica*, Folin–Ciocalteu colorimetry and the aluminum nitrate complex method were used to estimate the total phenolic content (TPC) and total flavonoid content (TFC) in CE and its four partition fractions. The TPC of CE and the four fractions were assessed by the equations of y = 0.0096x + 0.1279 (R² = 0.9977) for TPC and y = 0.0025x + 0.1005 (R² = 0.9994) for TFC according to the calibration curves of gallic acid and rutin, respectively. Table 1 shows that CE and its four fractions of *A. indica* contained a discrepant amount of TPC and TFC. The TPC level of the EA fraction (590.526 ± 1.468 mg GAE/g) was the highest, followed by the CE (541.111 ± 1.432 mg GAE/g) and n-BuOH fraction with 533.485 ± 2.143 mg GAE/g. Similarly, the TFC of EA fraction (280.800 ± 0.980 mg RE/g) was the highest, followed by CE with 180.667 ± 0.301 mg RE/g, and the n-Hex fraction was the lowest with 51.270 ± 0.366 mg RE/g. In addition, the TPC in n-Hex fraction (96.622 ± 0.779 mg GAE/g) was the lowest and was one sixth of TPC in EA fraction. Thus, this further explained that the EA fraction with the highest antioxidant activity could be ascribed to its high TPC and TFC content.

### 3.3. Screening for SOD and XOD Ligands in A. indica with UF-LC-MS

In order to further explore the responsible antioxidant components in the EA fraction, an ultrafiltration affinity screening with two targets (SOD and XOD) was conducted. As a result, nine and five compounds from the EA fraction of *A. indica* displayed different bindings to the SOD and XOD, respectively. The results of UF-LC/MS are listed in Table 2 for Rt (retention time), quasi-molecular ions ([M-H]^−^), characteristic MS/MS fragments and relative binding affinity (RBA), respectively. These compounds chosen by UF-LC-MS were identified by LC-ESI-MS/MS and UPLC-QTOF-MS/MS. 

**Figure 3:**
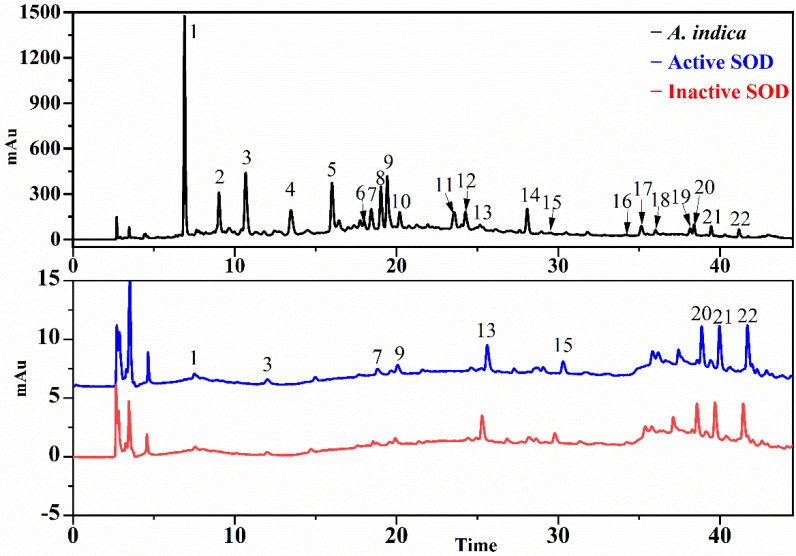
The UF-LC-UV chromatograms of EA fraction from *A. indica* with superoxide dismutase (SOD) at 280 nm. The black line represents the HPLC profiles of EA fraction, the blue and red line represent activated and inactivated SOD, respectively.

**Figure 4:**
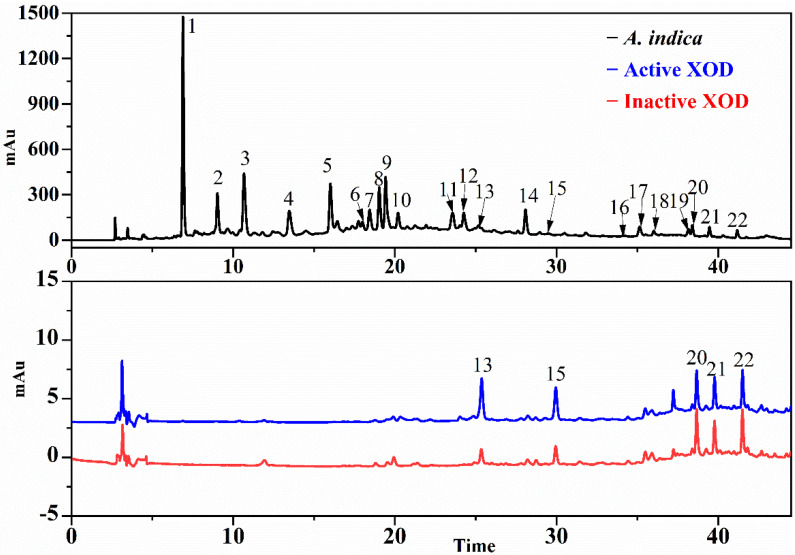
The UF-LC-UV chromatograms of EA fraction from *A. indica* xanthine oxidase (XOD) at 280 nm. The black line represents HPLC profiles of EA fraction, the blue and red lines represent activated and inactivated XOD, respectively.

As shown in Figure 3 and Figure 4, compounds in EA fraction exhibited different binding affinities to SOD and XOD. The differences in the peak areas between the active and inactive enzyme groups reflected differential binding affinity, namely, the potential ligands incubated with active enzymes displayed bigger peak areas than those of inactivated enzymes. In order to further evaluate the affinity between enzymes and ligands, the RBAs were calculated with following equation: RBA = Aa/Ab, where Aa and Ab represent the peak areas of the active and inactive enzyme group, respectively. Compounds were deduced to be well-founded as potential inhibitors of enzymes when the value of RBA was more than 1.5, and the values of RBA were sorted as follows: non-ligand less than 1.5, weak in the range of 1.5–2.0, moderate between 2.0 and 3.0 and strong when more than 3.0 [32]. 

Table 2 listed the RBA of potential ligands in EA fractions targeting SOD and XOD. For SOD, peak 1 displayed a moderate binding to SOD with the highest value of RBA at 2.15 > 2.0, followed by peaks 3 and 9 with the RBA values of 1.78 (>1.5) and 1.61 (>1.5), respectively; meanwhile, peak 7 (1.06), peak 13 (1.12), peak 15 (1.26), peak 20 (1.30) peak 21 (1.19) and peak 22 (1.29) showed a weak binding with RBA values less than 1.5. For XOD, peaks 13 exhibited a strong binding to XOD with the highest RBA value of 3.14, followed by peak 15, which showed a moderate binding with an RBA value of 2.05 > 2.0. Based on this, peaks 1, 3, and 9 were considered to be potential SOD ligands, and peak 13 and 15 were presumed to be XOD potential ligands for further study.

### 3.4. Identification of SOD and XOD Ligands in A. indica with UPLC-Q-TOF-MS/MS

In order to identify the ligands against SOD and XOD screened out above, UPLC-Q-TOF-MS/MS, in the negative-ion mode, was employed to characterize these ligands in the EA fraction. The structures of these compounds were assigned by the comparison with the MS/MS fragments reported in previous studies or public databases, such as PubChem or MassBank. In addition, retention times, fragment ions, and their identities are listed in Table 2. 

Peak 1 and peak 3 showed typical fragment ions at *m/z* 125.0253 and 109.0286, respectively. They were finally identified as gallic acid and protocatechuic acid [33,34] with a strong antioxidant activity [35,36,37] based on accurate mass and fragment ions, which suggests that peak 1 and peak 3 could contribute to the antioxidant potential of *A. indica*. Peak 7 ([M-H]^−^, *m/z* 593.1293) produced a base peak ion at *m/z* 423.0718 and the MS^2^ peaks of *m/z* 467.1010, 441.0726 and 305.0650. In this peak, the *m/z* 467.1010 was formed by the fracture of heterocyclic ring, and fragments *m/z* 423.0718 and 407.0664 were formed by RDA (retro-Diels–Alder reaction), and fragments *m/z* 305.0650 and 289.0655 were formed by quinone methide cracking. The RDA fragmentation of the top unit with a larger π-π hyperconjugated structure is more energetically favorable than the fragmentation of the base unit. Based on this, peak 7, with a secondary peak of *m/z* 289.0655, suggested that (epi)catechin was the top unit and (epi)gallocatechin was the base unit [38]. Peak 9 with the fragment ions at *m/z* 245.0879, 221.0811, 125.0197 and 109.0290 was identified as (−)-epicatechin possessing a prominent antioxidant activity [39,40]. The fragment ions at *m/z* 297.2421, produced by peak 13 ([M-H]^−^, *m/z* 459.2732), suggested that the presence of monohexoside through the loss of 162Da [41], but unfortunately, the final structure could not be identified solely by the MS data and existing information. Peak 15 ([M-H]^−^, *m/z* 331.1913) produced an MS^2^ fragment ion at *m/z* 287.1436 with the corresponding loss of the carboxylic acid group (44Da), and a fragment ion at *m/z* 244.0889 by the loss of the isopropyl radical from *m/z* 287.1436, which was classified as carnosic acid [42,43]. Peak 21 ([M-H]^−^, *m/z* 329.1757) yielded fragment ions at *m/z* 285.1050 by the loss of a carboxylic acid group (44Da) and were identified as carnosol by a comparison with the available literature [44,45]. Additionally, some studies have shown that carnosic acid and carnosol possessed a certain antioxidant activity [46,47].

**Table 2:** The relative binding affinity (RBA) and the UF-LC-MS data of potential SOD and XOD ligands in *A. indica*.

No.	Rt/min	[M-H]^−^ (*m/z*)	MS/MS Spectrum	Identification	RBA	
					SOD	XOD
1	7.29	169.0135	125.0253	Gallic acid [33]	2.15	ND
3	11.93	153.0189	109.0286	Protocatechuic acid [34]	1.78	ND
7	18.90	593.1293	467.1010, 441.0726, 423.0718, 407.0664, 305.0650, 289.0655, 245.0418, 125.0238	(epi)catechin-(epi)gallocatechin isomer [38]	1.06	ND
9	19.62	289.0716	245.0879, 221.0811, 205.0494, 203.0702, 125.0197, 109.0290	(−)-Epicatechin [39]	1.61	ND
13	24.31	459.2732	297.2421, 179.0339, 161.0228, 133.0281	Monohexoside derivatives [41]	1.12	3.14
15	28.11	331.1913	313.1792, 301.1781, 287.1436, 244.0889	Carnosic acid [43]	1.26	2.05
20	37.26	287.1658	255.1365, 199.0797, 186.0686	Unidentified	1.30	0.86
21	38.40	329.1757	285.1050, 269.1584, 245.0558, 213.0907	Carnosol [44,45]	1.19	0.93
22	40.24	285.1501	269.1133, 255.1002, 199.0743, 187.0725, 173.0576, 157.0664	Unidentified	1.29	0.83

“ND” Not detected.

### 3.5. Molecular Docking

**Figure 5:**
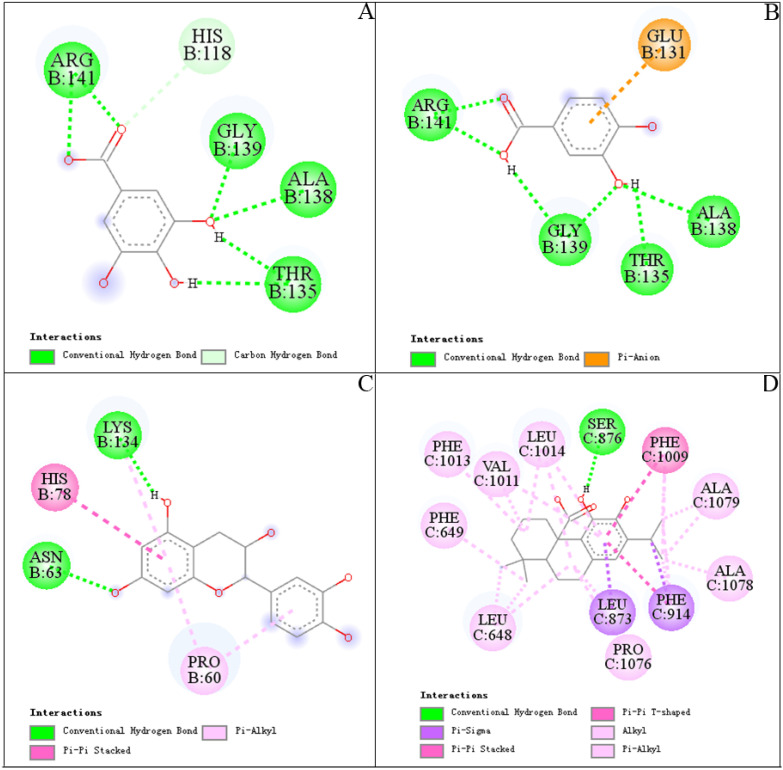
Docked complexes of SOD and XOD with peak 1 (gallic acid), peak 3 (protocatechuic acid), peak 9 ((−)-epicatechin) and peak 15 (carnosic acid) from *A. indica*: (**A**), SOD-gallic acid; (**B**), SOD-protocatechuic acid; (**C**), SOD-(−)-epicatechin; (**D**), XOD-carnosic acid. Blue lines represent hydrogen bonds.

After UF-LC-MS, three compounds (peak 1, 3, 9) and one compound (peak 15), as potential ligands for two target enzymes were docked with SOD and XOD according to the values of RBA, in order to further elucidate and predict the molecular interaction between ligands and target enzymes. The binding energy (BE), inhibition constant (Ki) and hydrogen bonds are summarized in Table 3, and their best docking conformations and binding sites are depicted in Figure 5. Dithiocarbamate (DTC) and allopurinol (ALL) were set as positive controls for SOD [48] and XOD [49], respectively. 

The Bes, shown in Table 3, were all below zero, indicating that the bindings of the compounds with SOD or XOD were energy-favorable reactions. Peak 9 ((−)-epicatechin) exhibited the strongest interaction with SOD (BE, −5.04 ± 0.01 kcal/mol; Ki, 203.14 ± 1.47 µM), followed by peak 3 (BE, −4.41 ± 0.01 kcal/mol; Ki, 588.63 ± 7.84 µM) and peak 1 (BE, −3.84 ± 0.37 kcal/mol; Ki, 1.69 ± 0.99 mM). Meanwhile, the BEs of peak 1 (gallic acid), peak 3 (protocatechuic acid) and peak 9 ((−)-epicatechin) were higher than positive control DTC (−2.77 ± 0.13 kcal/mol, 9.52 ± 2.05 mM). It is worth noting that peak 1 and peak 3 showed similar BEs, and they all formed six hydrogen bonds and the same amino acid residues, Arg141, Gly139, Ala138 and Thr135, with SOD in Figure 5A,B, which may be related to their similar structures. Hydrogen bonds were the main contribution to stabilizing the complex of gallic acid and protocatechuic acid. Inter-molecular interactions were also observed between peaks (1, 3) and SOD, including a carbon hydrogen bone and pi-anion. From Figure 5C, two hydrogen bonds were formed between hydroxyl groups of (−)-epicatechin and amino acid residues (Lys134, Asn63) of SOD. Pi-alkyl (Pro60) and pi–pi Stacked (His78) interactions were also the force that kept (−)-epicatechin-SOD complex stable. When compared with the positive control ALL (−6.28 ± 0.00 kcal/mol, 24.82 ± 0.00 µM), peak 15 (carnosic acid) showed a higher ability binding to XOD with a BE of -8.24 ± 0.71 kcal/mol and Ki of 1.27 ± 1.24 µM. Figure 5D showed that carnosic acid contacted XOD by hydrogen bonds with Ser 876, pi–pi with Phe1009, Pi-sigma with Leu873 and Phe914. In addition, amino acid residues of XOD, including Phe1013, Phe649, Leu1014, Leu648, Val1011, Ala1078, Ala1079 and Pro1076, also interacted with carnosic acid through alkyl or pi-alkyl. Meanwhile, the crystal coordinates of 2-hydroxybenzoic acid under the access code 1FIQ were used to calculate RMSD, which is 1.8Å (value lower than 2Å for successful docking results).

**Figure 6:**
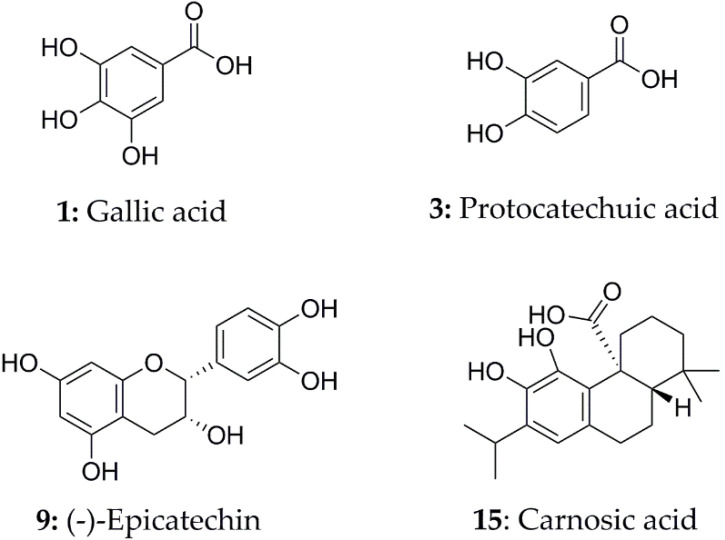
The potential ligands screened from EA fraction of *A. indica* by UF-LC-MS with superoxide dismutase (SOD) and xanthine oxidase (XOD).

In summary, the BEs of peaks 1, 3, 9 and 15 were superior to the positive controls, theoretically indicating that the peaks had a high inhibition effect on SOD and XOD, respectively. Hence, peak 1 (gallic acid), peak 3 (protocatechuic acid), peak 9 ((−)-epicatechin) for SOD and 15 (carnosic acid) for XOD were identified as potential ligands for further study. Their structures are shown in Figure 6, the retention times are shown in Figures S2 and S3 and the mass spectrometry fragments are shown in Figures S4−S7.

### 3.6. Antioxidant Capacity of Potential Ligands by UF-LC-MS

**Figure 7:**
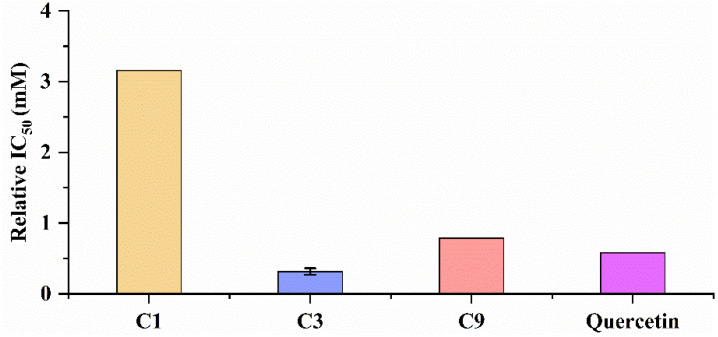
The relative IC_50_ of potential ligands with SOD. C1: gallic acid; C3: protocatechuic acid; C9: (−)-epicatechin.

According to the UF-LC-MS, the relative IC_50_ of potential ligands with SOD was evaluated, and the result is shown in Figure 7 and UF-LC-MS is shown in Figure S1. As shown in Figure 7, compound 3 (protocatechuic acid, IC_50_ = 0.31513 ± 0.04581) showed the strongest activity to SOD, followed by (−)-epicatechin and gallic acid.

## 4. Conclusions

It was reported that *A. indica* showed a wide spectrum of pharmacological activities, such as antioxidant, diuretic, liver protection, and anticancer activities. Currently, the active ingredients responsible for its antioxidant or antigout activity remain unexplored. Taking advantage of the fact that both SOD and XOD are effective targets for the treatment of diseases related to oxidative damage, we managed to develop an integrated strategy combining bioaffinity ultrafiltration with SOD and XOD coupled with LC-MS/MS in order to screen its active antioxidant ingredients from the EA fraction, which showed a better antioxidant activity and higher contents of TFC and TPC than those of other fractions from *A. indica*. As a result, peaks 1 (gallic acid), peak 3 (protocatechuic acid) and peak 9 ((−)-epicatechin) were presumed to be potential ligands of SOD, and peak 15 (carnosic acid) was identified as a potential ligand of XOD based on the RBA values from UF-LC/MS. Then, a molecular docking analysis was used to predict the interactions between these four ligands and two target enzymes, and these four compounds, corresponding to peak 1, 3, 9 and 15, were even more superior to the positive controls, which were in good agreement with the results obtained by UF-LC/MS with SOD and XOD. In conclusion, this study showcased an integrative strategy to selectively screen out the promising natural antioxidants in the EA fraction from *A. indica* by UF-LC-MS with multiple target enzymes (such as SOD and XOD), which could facilitate not only the exploration of its potential responsible active compounds in *A. indica*, but also offers a useful guidance for screening out other active compounds from other medicinal plants of high interest. Meanwhile, it is worth stating that the results only reveal potential effects at this research stage, and further experiments are still needed to determine the corresponding therapeutic effects in the later stage. From another perspective, gout patients are often accompanied by oxidative damage. In this study, XOD is not only a target related to antioxidant activity, but also a classical target for the treatment of gout. On the basis of antioxidant active components of *A. indica*, the method developed in this study can also be used to rapidly discover potential multi-target active compounds against gout from *A. indica*, so as to explore the relationship between oxidative damage and gout, and explore their mechanisms of action.

## Figures and Tables

**Table 1:** The contents of total polyphenols and total flavones of *A. indica*.

Samples	TPC (mg GAE/g dw)	TFC (mg RE/g dw)
CE	541.111 ± 1.432 ^b^	180.667 ± 0.301 ^b^
n-Hex	96.622 ± 0.779 ^e^	51.270 ± 0.366 ^e^
EA	590.526 ± 1.468 ^a^	280.800 ± 0.980 ^a^
n-BuOH	533.485 ± 2.143 ^c^	89.333 ± 0.562 ^c^
WA	287.583 ± 0.745 ^d^	69.387 ± 0.261 ^d^

Mean values with different letters (a, b, c, d and e, respectively) were significantly different (*p* < 0.05, ANOVA, DMRT, *n* = 3) in the columns. TPC, total phenolic content; TFC, total flavonoids content; GAE/g dw, gallic acid equivalent per gram of dry weight; RE/g dw, rutin equivalent per gram of dry weight.

**Table 3:** Docking ability and affinity of potential ligands with SOD and XOD.

Peaks	SOD (PDB 1CBJ)			XOD (PDB 1FIQ)		
	BE (Kcal/mol)	Ki	Hydrogen Bonds	BE (Kcal/mol)	Ki	Hydrogen Bonds
1	−3.84 ± 0.37	1.69 ± 0.99 mM	Arg141, Gly139, Ala138, Thr135	ND	ND	ND
3	−4.41 ± 0.01	588.63 ± 7.84 µM	Arg141, Gly139, Thr135, Ala138	ND	ND	ND
9	−5.04 ± 0.01	203.14 ± 1.47 µM	Lys134, Asn63	ND	ND	ND
15	ND	ND	ND	−8.24 ± 0.71	1.27 ± 1.24 µM	Ser 876
DTC ^#^	−2.77 ± 0.13	9.52 ± 2.05 mM	His61, Asn63	ND	ND	ND
ALL ^##^	ND	ND	ND	−6.28 ± 0.00	24.82 ± 0.00 µM	Phe1009, Ala1149

BE, binding energy; Ki, inhibition constant; DTC, dithiocarbamate; ALL, Allopurinol; ^#^, positive control of SOD; ^##^, positive control of XOD; ND, not detected. Abbreviations of glycosyl amino acid residues: Arg, arginine; Gly, glycine; Ala, alanine; Thr, threonine; Lys, Lysine; Asn, asparaginate; His, histidine; Ser, serine; Phe, phenylalanine.

## Data Availability

All data in this study are included in this article and supplementary materials.

## Supplementary Materials

The following supporting information can be downloaded at: https://www.mdpi.com/article/10.3390/antiox11040658/s1, Figure S1: The UF-LC-MS of the potential ligands of SOD, Figure S2: The HPLC of the potential ligands of SOD, Figure S3: The HPLC of the potential ligands of XOD, Figure S4: The mass spectrometry fragments of gallic acid in standard substance and *A. indica*, Figure S5: The mass spectrometry fragments of protocatechuic acid in standard substance and *A. indica*, Figure S6: The mass spectrometry fragments of (−)-Epicatechin in standard substance and *A. indica*, Figure S7: The mass spectrometry fragments of carnosic acid in standard substance and *A. indica*, Table S1: The relative binding affinity (RBA) and the relative IC_50_ data of potential SOD ligands in *A. indica*.
