# Supplementary material for: Potential Antioxidative Components in Azadirachta indica Revealed by Bio-Affinity Ultrafiltration with SOD and XOD
Source: Antioxidants (Basel). 2022 Mar 29;11(4):658. doi: 10.3390/antiox11040658 (PMC9030372; doi:10.3390/antiox11040658)
Supplement: Supplementary file 1 [file antioxidants-11-00658-s001.zip › antioxidants-1611120-supplementary.pdf]

antioxidants

Supplementary materials

Mingquan GUO
Professor of Medicinal Chemical Biology
Wuhan Botanical Garden, Chinese Academy of Sciences
Email: guomq@wbgcas.cn
TEL./FAX.: +86-(027)87700850

March 22, 2022

Potential Antioxidative Components in *Azadirachta indica* Revealed by Bio-affinity Ultrafiltration with SOD and XOD

Minxia Fan [1, 2, 3], Guilin Chen [1, 2, 3], and Mingquan Guo [1, 2, 3, *]

[1] Key Laboratory of Plant Germplasm Enhancement and Specialty Agriculture, Wuhan Botanical Garden, Chinese Academy of Sciences, Wuhan 430074, China; fanminxia@wbgcas.cn (M.-X.F.); glchen@wbgcas.cn (G.-L.C.)
[2] Sino-Africa Joint Research Center, Chinese Academy of Sciences, Wuhan 430074, China
[3] Innovation Academy for Drug Discovery and Development, Chinese Academy of Sciences, Shanghai 201203, China
* Correspondence: guomq@wbgcas.cn; Tel.: +86-027-87700850

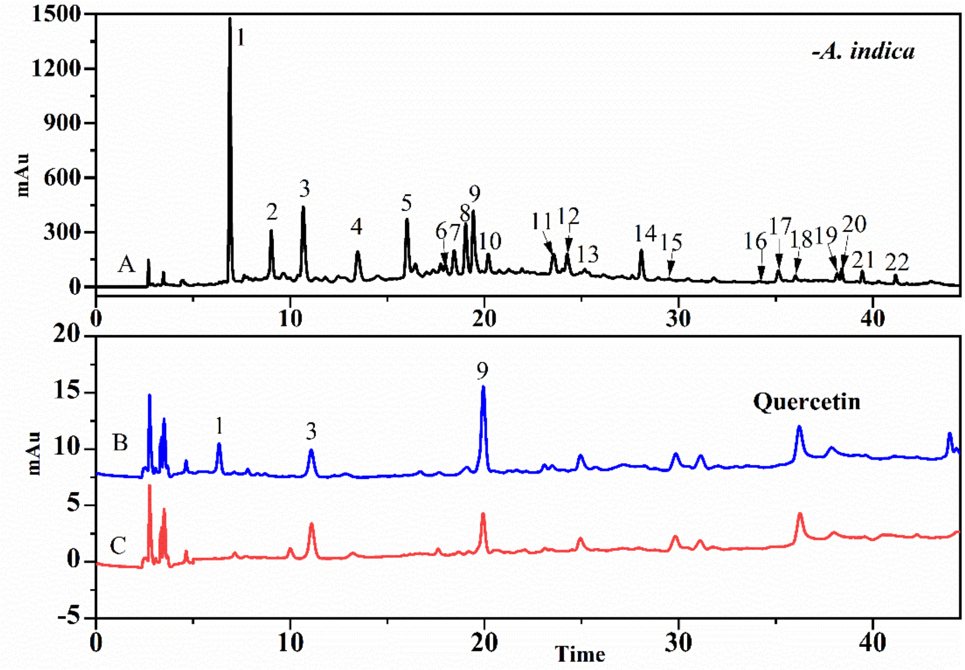

Figure S1. The UF-LC-MS of the potential ligands of SOD. Line A: *A. indica;* Line B: UF-LC-MS of Gallic acid+Protocatechuic acid+(-)-Epicatechin+Quercetin with actice SOD; Line C: UF-LC-MS of Gallic acid+Protocatechuic acid+(-)-Epicatechin+Quercetin with inactice SOD; 280nm

Table S1. The relative binding affinity (RBA) and the relative IC_{50} data of potential SOD ligands in *A. indica*.

No.	Identification	RBA	IC_{50} (mM)
		SOD	
1	Gallic acid	0.084	3.15
3	Protocatechuic acid	0.84	0.32
9	(-)-Epicatechin	0.34	0.79
Quercetin		0.46	0.58

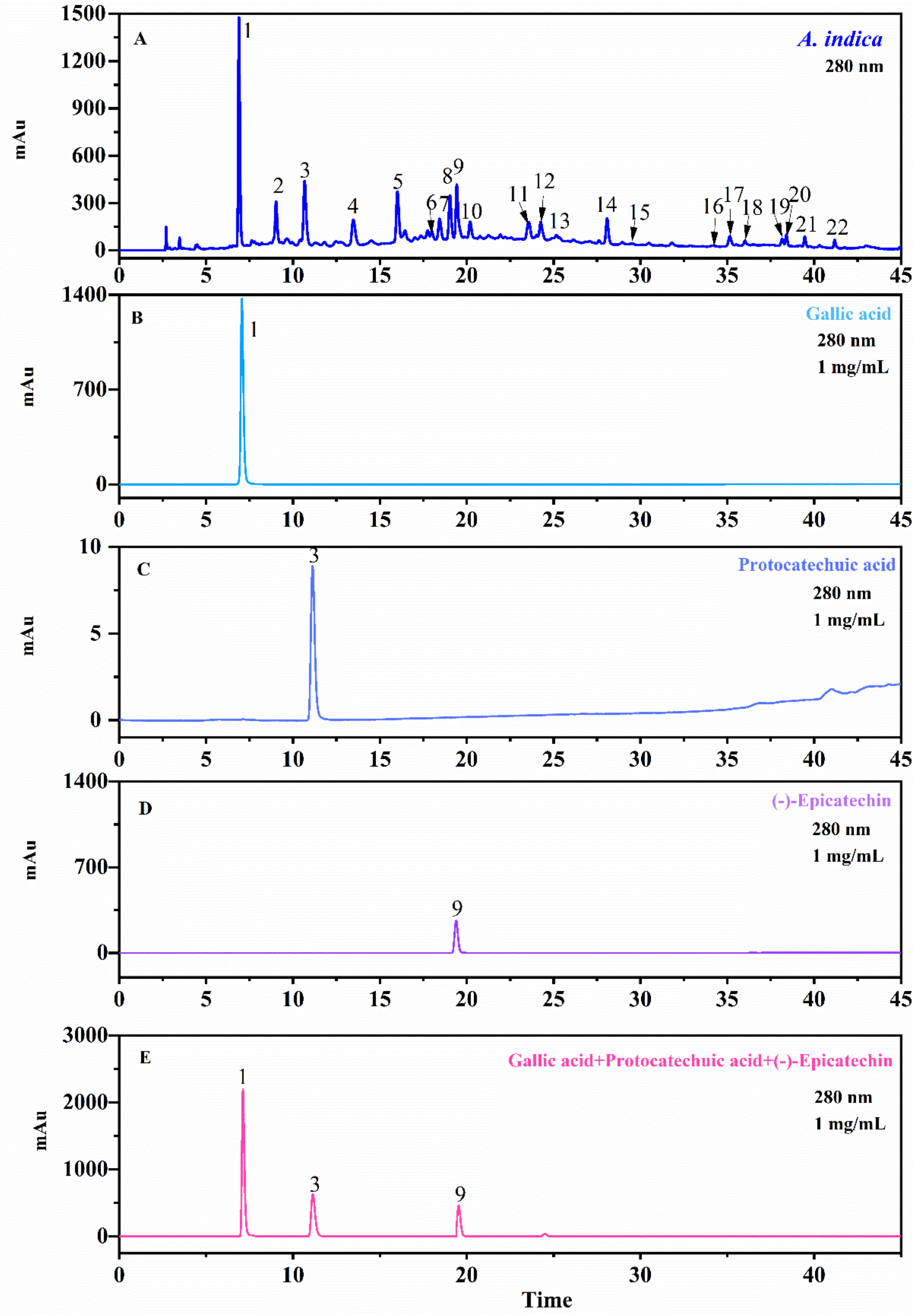

Figure S2. The HPLC of the potential ligands of SOD. A: *A. indica*; B: Gallic acid; C: Protocatechuic acid; D: (-)-Epicatechin; E: Gallic acid+Protocatechuic acid+(-)-Epicatechin

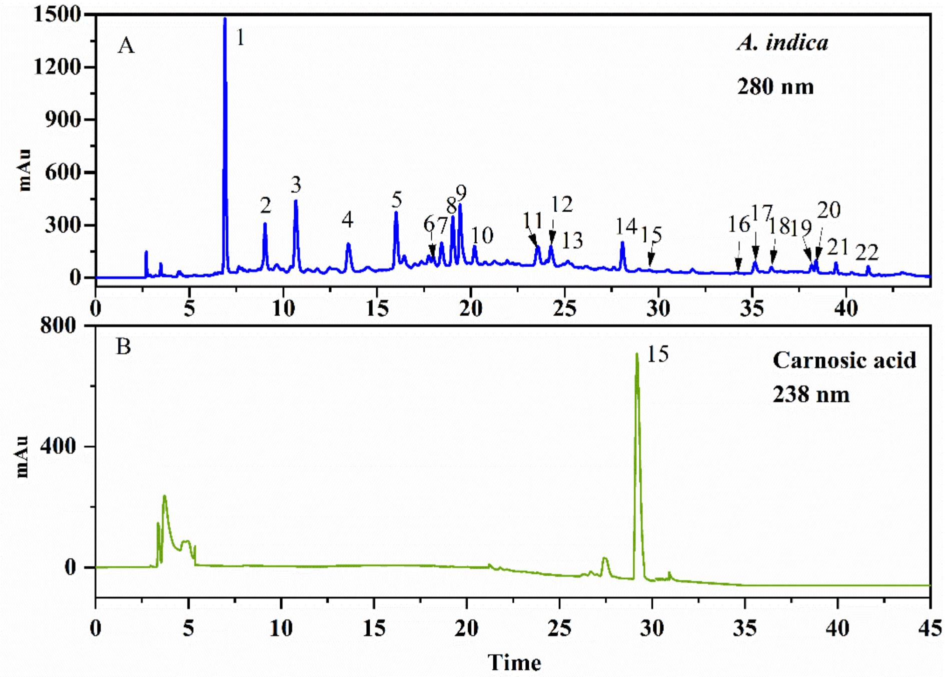

Figure S3. The HPLC of the potential ligands of XOD. A: *A. indica*; B: Carnosic acid.

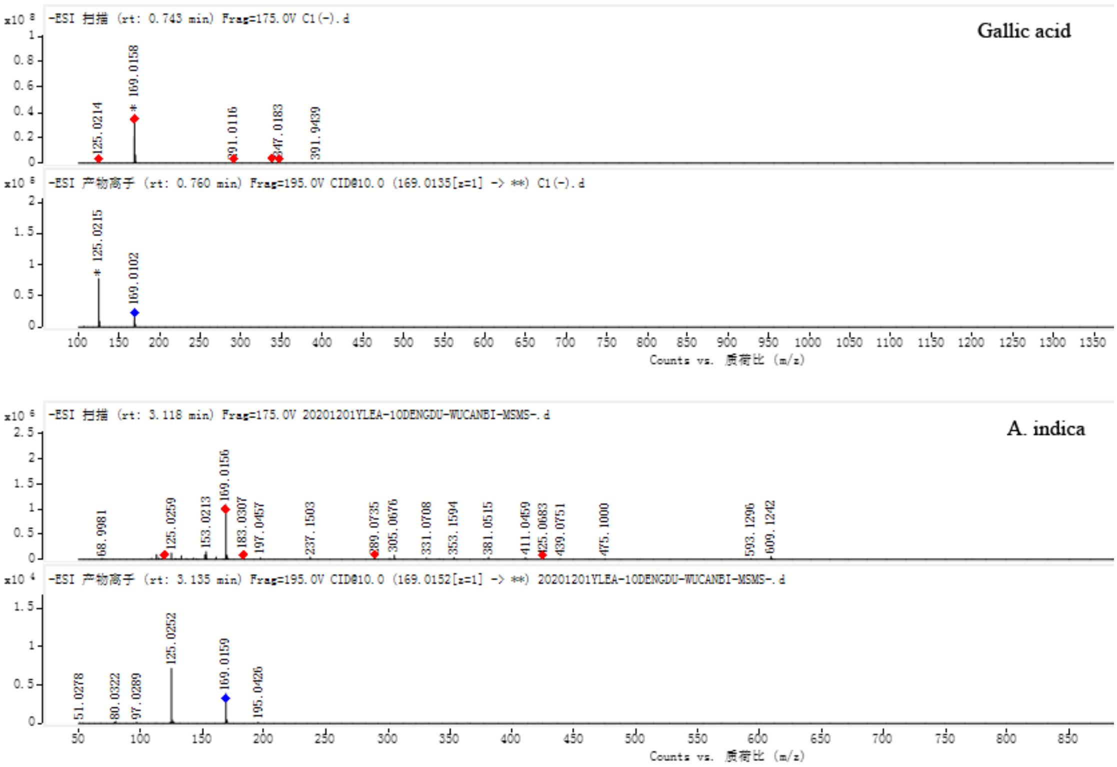

Figure S4. The mass spectrometry fragments of gallic acid in standard substance and *A. indica*.

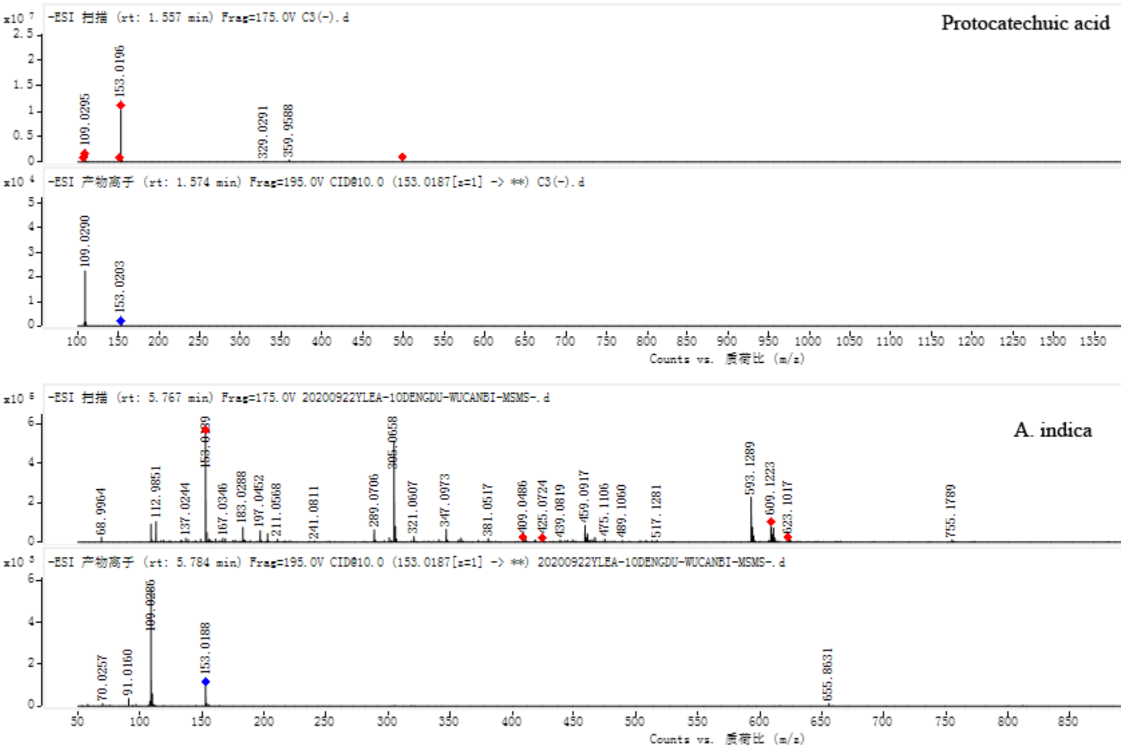

Figure S5. The mass spectrometry fragments of protocatechuic acid in standard substance and *A. indica*.

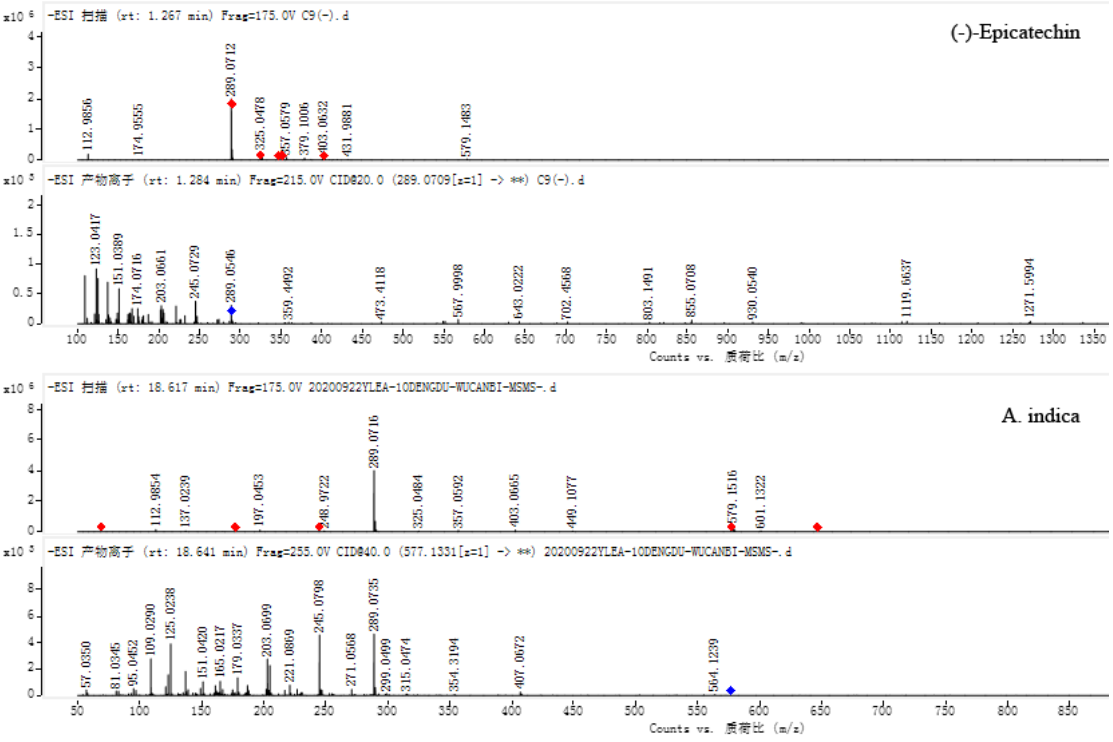

Figure S6. The mass spectrometry fragments of (-)-Epicatechin in standard substance and *A. indica*.

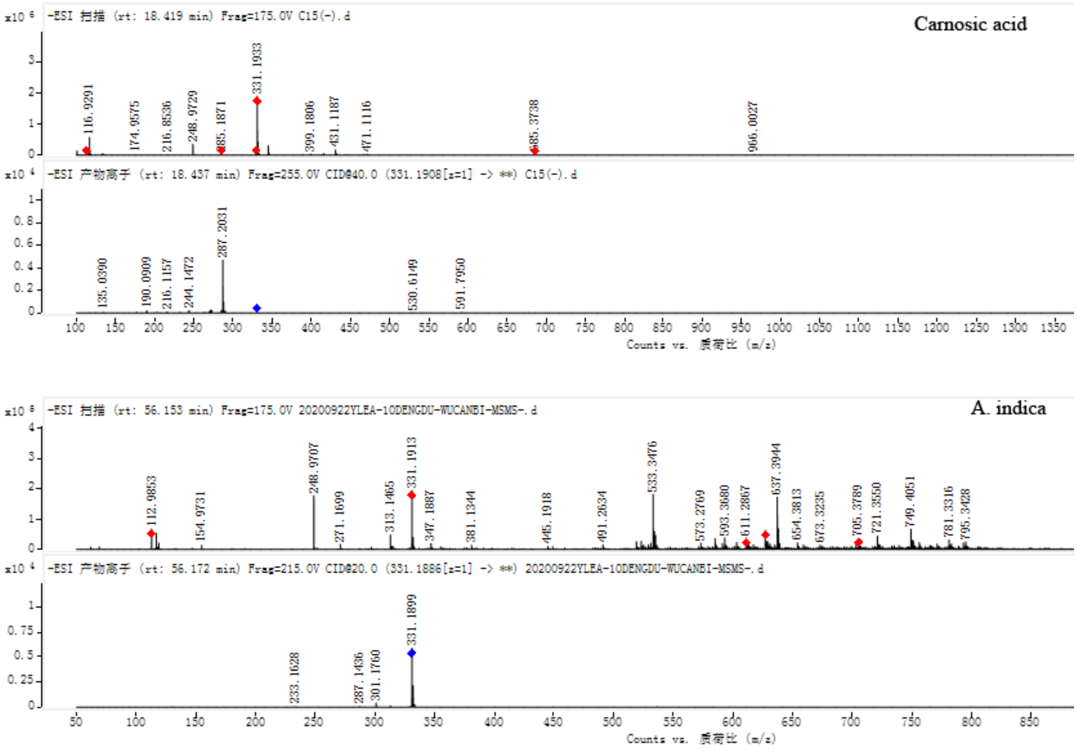

Figure S7. The mass spectrometry fragments of carnosic acid in standard substance and *A. indica*.
